# Urban change detection: assessing biophysical drivers using machine learning and Google Earth Engine

**DOI:** 10.1007/s10661-025-13863-4

**Published:** 2025-03-20

**Authors:** Olufemi Sunday Durowoju, Rotimi Oluseyi Obateru, Samuel Adelabu, Adeyemi Olusola

**Affiliations:** 1 Department of Geography, University of the Free State, Bloemfontein Campus, Bloemfontein, South Africa; 2 Department of Geography, Osun State University, Okuku Campus, Okuku, Nigeria; 3 Department of Geography, University of Ibadan, Ibadan, Oyo State Nigeria; 4 Faculty of Environmental and Urban Change, York University, Toronto, ON Canada

**Keywords:** Landscape changes, Land use and land cover, Random forest, Support vector machines, Urban ecosystem

## Abstract

Urban areas are experiencing rapid transformations, driven by population growth, economic development, and policy changes. Understanding and monitoring these dynamic changes is crucial for sustainable urban planning and management. This study leverages machine learning and Google Earth Engine to investigate urban dynamics and its interactions with biophysical conditions in the Kaduna River Basin (KRB), Nigeria. This study utilized a dataset of 192 points, initially extracted from Google Earth Engine, to analyze urban transitions between 1987 and 2020, incorporating biophysical and environmental variables such as population density, precipitation, and surface temperature. The dataset was processed in R using the CARET package, with missing data imputed via K-nearest neighbors (KNN), categorical variables transformed using One-Hot Encoding, and numerical variables rescaled for consistency. A tenfold cross-validation approach was used to train and validate machine learning models, including random forest, support vector machine, KNN, and multivariate adaptive regression splines, ensuring optimal model performance. Model evaluation metrics such as overall accuracy, kappa, F1 score, and area under the curve confirmed the reliability of the models in identifying the biophysical factors influencing urban changes. The findings revealed overall accuracy of 0.80, 0.73, 0.71, and 0.72 and kappa statistics of 0.60, 0.46, 0.42, and 0.45 for random forest (RF), multivariate adaptive regression splines, support vector machine, and KNN, respectively, with RF emerging as the most accurate model (80%) for predicting urban change patterns in KRB. Land cover changes reveal rapid urban expansion (154.81%), declining water bodies (− 95.79%), and vegetation growth (174%). Machine learning models identify population density and water stress index as key urban change drivers, with climate factors like temperature and precipitation playing crucial roles. The results of this study offer valuable insights into the processes driving urban transformation and present a robust methodology for monitoring and predicting future urban development. This study aids in the creation of strategies for sustainable urban growth and the mitigation of adverse environmental impacts.

## Introduction

Understanding the dynamics of urban change, its transitions, and landscape risk evaluation in river basins is critical for sustainable urban planning and resilience building. As global urbanization accelerates, with one-third of land use changing at least once over recent decades (Winkler et al., 2021), the need for advanced monitoring and predictive tools is more pressing than ever. This urban expansion, driven by population growth, economic development, and policy shifts, poses significant challenges for water resources, food security, and climate resilience (Song et al., 2018; Winkler et al., 2021). Furthermore, land use change plays a crucial role in shaping ecosystems and environmental health, influencing factors such as water availability, soil quality, and biodiversity (Obateru et al., 2024). As the global urban population is expected to reach 61% by 2030, with much of the growth concentrated in developing regions like Africa and Asia (Onyango, 2018), it is essential to understand how urbanization interacts with biophysical processes in rapidly growing regions.

Recent studies have shown that indirect factors, such as climate change, account for about 40% of global urban changes, with the remaining 60% driven by direct human activities (Chai et al., 2022; He et al., 2024; Li et al., 2021; Song et al., 2018; van Vliet, 2019; World Cities Report, 2024). These changes vary regionally, with land use activities driving urban transformation in Europe, South America, Asia, and Africa (Song et al., 2018). For instance, sub-Saharan Africa has experienced significant deforestation, linked to both smallholder agriculture and commercial crop cultivation (Ordway et al., 2017). Meanwhile, in the savannahs of Central and West Africa, increasing precipitation and carbon dioxide have led to forest expansion and woody encroachment, reshaping regional landscapes (Mitchard & Flintrop, 2013). In West Africa, the consequences of urbanization on biophysical variables such as precipitation, runoff, soil moisture, and vegetation are particularly pronounced. These changes disrupt ecosystem services such as carbon sequestration, water regulation, and biodiversity conservation, impacting climate change mitigation and adaptation strategies (Fashae et al., 2017; Liu et al., 2019; Polasky et al., 2011). As urbanization accelerates, the vulnerability of urban and peri-urban populations to climate change intensifies, making it crucial to identify emerging urban spaces and develop effective policy responses that address both environmental and socio-economic challenges.

This study aims to investigate urban change dynamics in the Kaduna River Basin, Nigeria, focusing on how urban transformations influence critical biophysical variables such as water availability, land cover, and ecosystem services. A key contribution of this work is its use of advanced spatial techniques, including machine learning algorithms (MLAs) and Google Earth Engine (GEE), to analyze urban transitions between 1987 and 2020. Unlike earlier studies, which often relied on traditional spatial analysis, this research leverages the power of cloud computing and geospatial data to identify and predict changes with greater accuracy and speed. This study employs a novel methodological approach that integrates expert knowledge with spatial analysis and machine learning algorithms to refine data selection, enhance model interpretation, and improve classification accuracy. By leveraging domain expertise, this approach ensures a deeper understanding of urbanization’s impact on biophysical conditions, leading to more informed and contextually relevant insights for sustainable urban planning strategies.

The implications of this study extend far beyond the basin of interest. It highlights the broader relevance of integrating machine learning, remote sensing, and biophysical data to understand urban dynamics. Machine learning techniques such as random forest (RF), support vector machine (SVM), K-nearest neighbor (KNN), and multivariate adaptive regression splines (MARS) have proven to be powerful tools for classifying and predicting land use and land cover (LULC) changes (Amani et al., 2020; Kang & Kanniah, 2022). Using computing platforms like Google Earth Engine allows for the rapid processing of extensive spatial datasets, making it possible to detect and analyze urban changes even in data-scarce regions (Sun et al., 2021; Tankpa et al., 2020). This approach underscores the global potential of such methodologies to monitor urban transformations and their environmental impacts, enabling researchers and policymakers to address urban sustainability challenges effectively. The integration of biophysical variables into these analyses not only enhances our understanding of how urbanization alters ecosystems but also provides a pathway for data-driven decision-making in managing urban growth across diverse contexts, particularly in rapidly developing regions.

## Materials and methods

### Study area

**Fig. 1:**
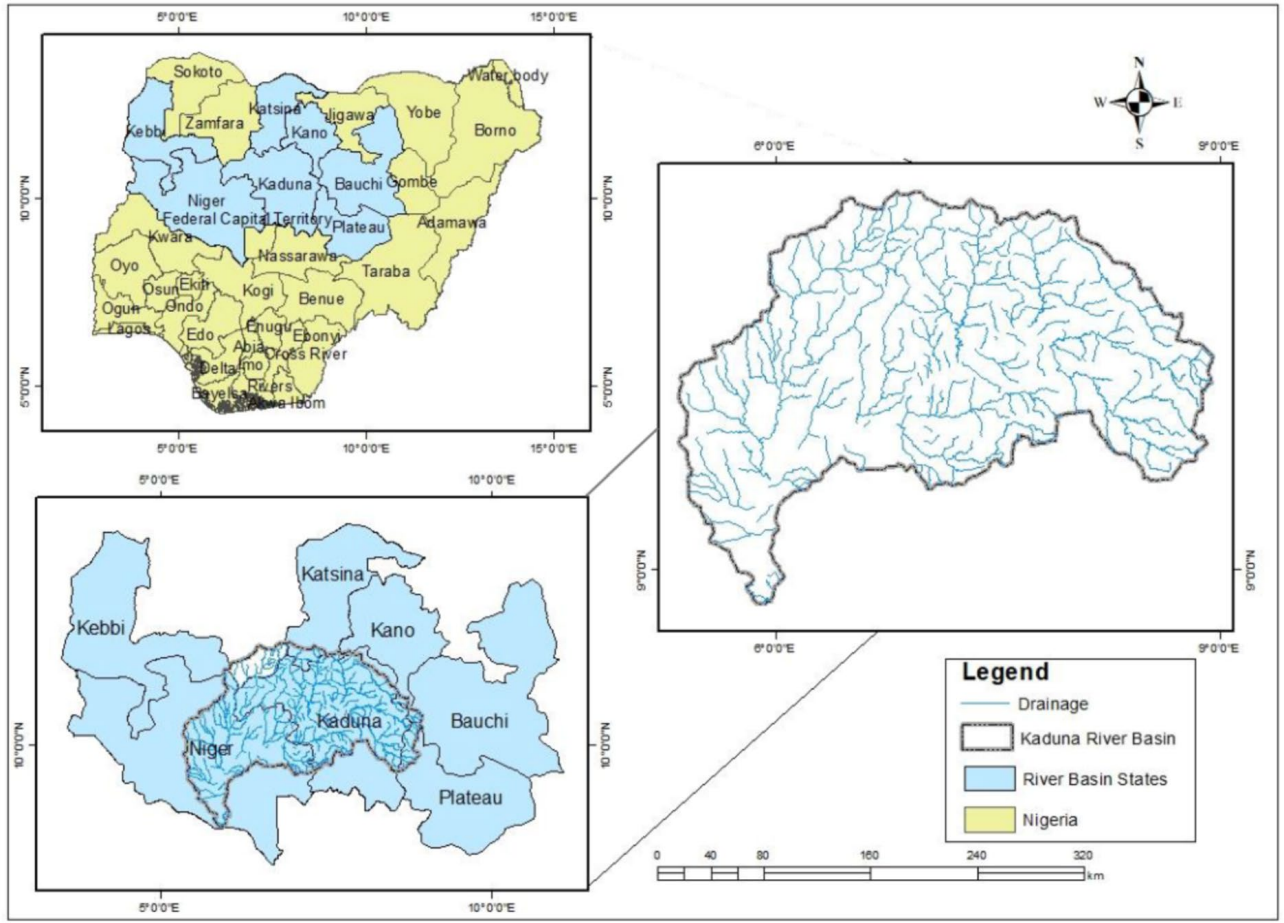
Map of the study area

The research was conducted in the Kaduna River Basin (KRB) (Fig. 1). It is strategically located between latitudes 8°45′15″N and 11°40′5″N and longitudes 5°25′48″E and 8°45′36″E in the savanna ecological zone of Nigeria. KRB has a catchment area of approximately 65,878 km^2^, with its headwater near the northeastern edge of the Jos Plateau at Sherri Hills. Shiroro Reservoir (320 km^2^) is situated within the study area to supply energy to the country and neighboring countries (Durowoju et al., 2022). The prevailing climate over the basin is described as a tropical continental climate (Aw) characterized by distinct wet and dry seasons, resulting in seasonal variations in rainfall and temperature patterns (Koppen, 1928). The mean annual precipitation can range from exceptionally high levels of 2000 mm during wet years to as low as 500 mm in drought years, but it typically maintains a long-term average of 1000 mm, with an average annual temperature of approximately 27.48 °C (Durowoju et al., 2022). The basin has become one of Nigeria’s focused regions for urbanization and economic development (Durowoju et al., 2021a, 2023). Over the past two decades, Kaduna River Basin’s population grew from about 14.3 million to about 27.3 million at an average annual growth of 4.48% and a population density of 19 per km^2^ (worldometers.info/world-population/niger-population).

### Methods

**Fig. 2:**
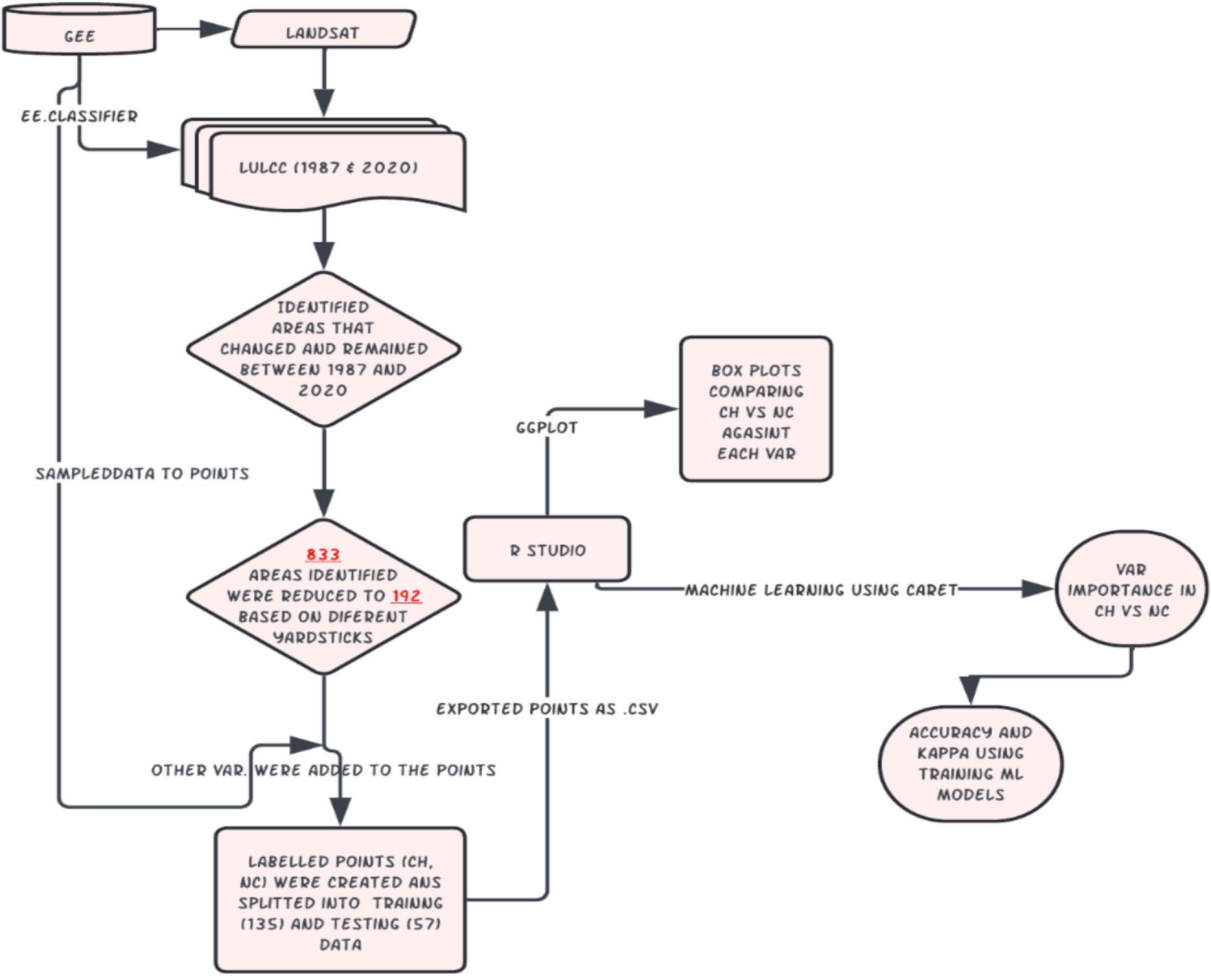
Workflow of the methodology

The dataset used in this study was initially prepared by exporting points from Google Earth Engine (GEE) (Fig. 2). The key objective was to identify urban areas that had either transitioned or remained unchanged between 1987 and 2020. This was achieved by analyzing land cover classifications derived from Landsat 5 Thematic Mapper (TM) (30-m resolution) for 1987 and Landsat 8 Operational Land Imager (OLI) (30-m resolution) for 2020. By comparing areas classified as urban settlements in 1987 with those in 2020, the study was able to quantify patterns of urban expansion and stability over time. Around 800 points were initially identified as either transitioning or maintaining their urban status. These points were classified as either “changed” (CH) or “unchanged” (NC), and a subset of 192 points was selected based on expert knowledge and field experience (Durowoju, 2021), with the final classification controlled for factors such as extent, coverage, and accuracy (Fig. 2). To further understand how urban changes could impact the biophysical functioning of the region and the provision of ecosystem services, a set of environmental and biophysical variables was extracted for the 192 selected points (Table 1). These variables included population density, leaf area index (LAI), precipitation, total runoff, surface runoff, potential evapotranspiration (PET), surface temperature, solar radiation, latent heat flux, volumetric soil water content, soil temperature, and a derived Water Stress Index (WSI), which was calculated using the formula:

**Table 1:** Selected variables

Variables	Unit	Abbreviation	Source
Potential evapotranspiration	m	PE	ERA5
Total runoff	m	Trunoff	ERA5
Surface runoff	m	Srunoff	ERA5
Total evaporation	m of equivalent water	TE	ERA5
Total precipitation	m	TP	ERA5
Temperature	°C	Temp	ERA5
Leaf area index	m^2^ m^−2^	LAI	ERA5
Surface net solar radiation	J/m^2^	SR	ERA5
Soil temperature	°C	STemp	ERA5
Latent heat flux	J/m^2^	LHF	ERA5
Volumetric soil water layer	m^3^ m^−3^	VSW	ERA5
Population density	people/km^2^	pop	NASA Socioeconomic Data and Applications Center (SEDAC)
Water Stress Index	-	WSI

1 $$Water\;stress\;index\;=\;\;Total\;runoff/Population\;density$$

The dataset (192 points and the biophysical variables) was imported into R (Team, R-Core 2021), where various machine learning models were applied using the CARET package (Kuhn, 2008). This methodology explored the underlying biophysical factors that may influence urban changes. The dataset was divided into training and testing subsets using a 70–30 split, ensuring robust model validation and mitigating overfitting. Data preparation involved several crucial steps. First, any missing data was imputed using the K-nearest neighbors (KNN) method. This was done by applying the *preProcess* function with the “knnImpute” method from the CARET package. Imputation ensured that the dataset was complete and ready for further analysis. Following imputation, One-Hot Encoding was used to convert categorical variables into binary dummy variables. This step ensured that the machine learning algorithms could effectively interpret and utilize the categorical data. The *dummyVars* function from CARET facilitated this transformation. The entire dataset was then rescaled to account for variations in the scale and units of the explanatory variables. This rescaling was performed using the *preProcess* function with the “range” method, which standardized the variables to a common scale. Such standardization is critical for machine learning models as it ensures that differences in the magnitude of variables do not unduly influence the analysis. Hyperparameter tuning and cross-validation were essential components of the model training process.

A tenfold cross-validation technique was employed using the *trainControl* function from CARET. This approach enabled a thorough evaluation of model performance and helped prevent overfitting by ensuring that the models generalized well to unseen data. Several machine learning algorithms were selected for their ability to handle complex relationships within the data. The algorithms included random forest (RF), support vector machine (SVM), KNN, and multivariate adaptive regression splines (MARS) (Cover & Hart, 1967; Breiman, 2001; Friedman, 1991; Cortes &Vapnik, 1995). Each algorithm was trained on the prepared training dataset (70%), with the process tailored to optimize model performance based on the defined hyperparameters. The trained models were then evaluated on the test dataset (30%). Key evaluation metrics included overall accuracy (OA), kappa, F1 score, and area under the curve (AUC). These metrics provided a comprehensive assessment of model performance, capturing various classification accuracy and robustness aspects. The methodology outlined above ensured that the analysis was rigorous and that the resulting models were accurate and generalizable, providing valuable insights into the biophysical factors influencing urban changes.

## Results

### Biophysical variables and urban change

**Fig. 3:**
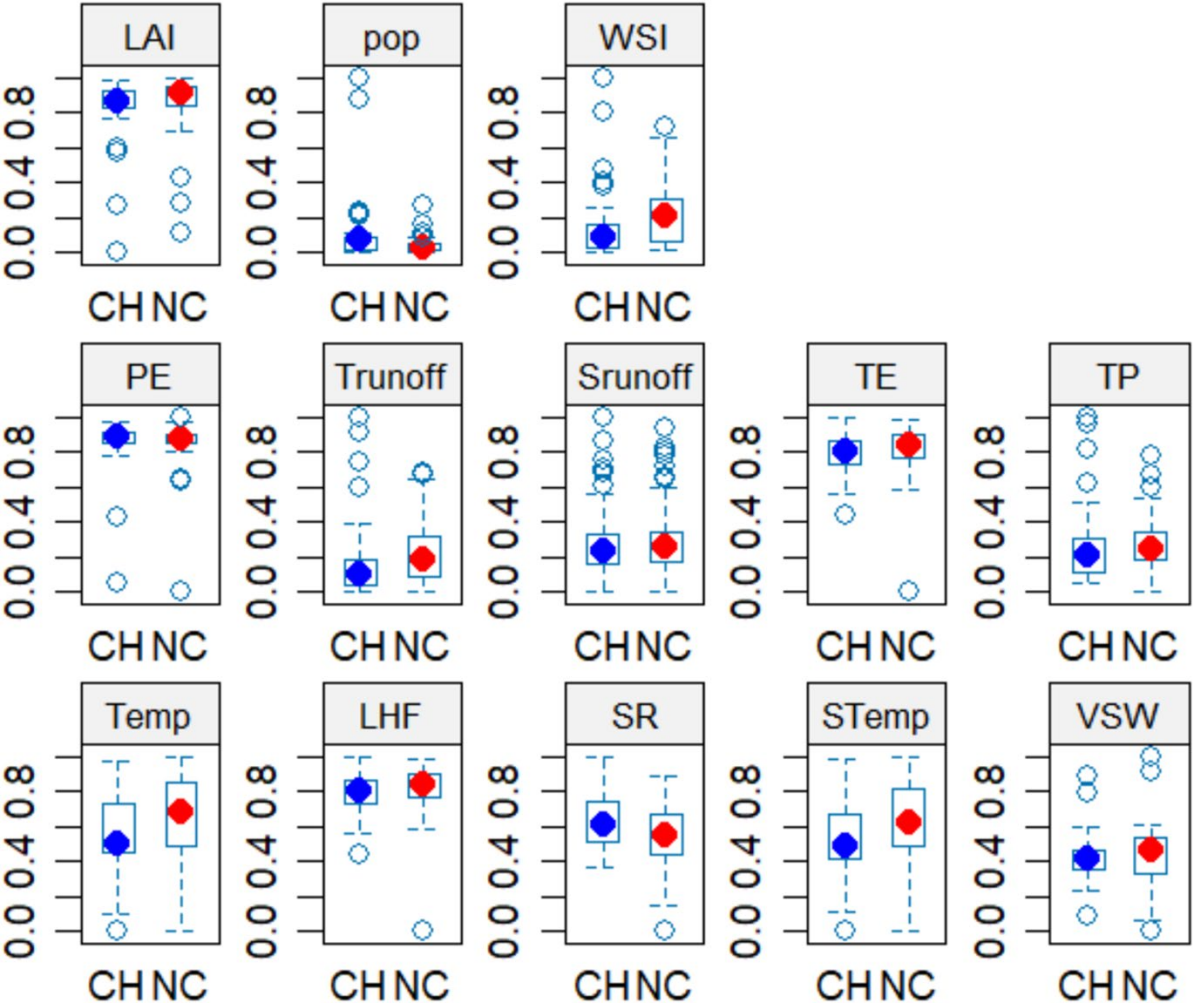
Boxplots showing the distribution of changes within the Kaduna Basin

Figure 3 shows how the selected biophysical variables discriminate between urban changes. As urban areas within the basin evolve, both in structure and function, these variables serve as important predictors or are significantly affected by urbanization (Ma et al., 2010; Sharma et al., 2013). Understanding the interplay of these factors is crucial for urban planning, environmental management, and sustainable development. These biophysical variables highlighted in Fig. 3 are integral to understanding and managing the impacts of urbanization. They provide valuable insights into water cycle dynamics, hydrological responses, temperature regulation, ecological health, and socio-economic challenges. By monitoring and analyzing these variables, urban planners and policymakers can develop strategies to promote sustainable urban development, enhance resilience to environmental changes, and improve the quality of life for urban residents.

Specifically, the boxplots highlight significant differences in median values, spread, and interquartile ranges (Fig. 3), illustrating urbanization’s impact on environmental and climatic conditions within the Kaduna River Basin (KRB). These changes reflect the complex relationships between land-use changes, hydrological processes, and atmospheric dynamics, shaped by the basin’s tropical continental climate and diverse land cover (Durowoju et al., 2022). Key differences between urbanized areas (CH) and non-urbanized areas (NC) include water cycle variables. Potential evapotranspiration (PE) is slightly higher in CH areas, indicating greater atmospheric water demand. In contrast, total runoff is significantly higher in NC areas due to the landscape’s ability to generate surface flow, particularly in deforested regions (Song et al., 2018). Surface runoff remains similar across both areas, suggesting that urbanization has not drastically altered immediate drainage characteristics. Precipitation-related processes also show variations, with total evaporation (TE) and total precipitation (TP) higher in NC areas, indicating their effectiveness at retaining water (Oke, 1982). Interestingly, temperatures at 2 m are higher in NC areas, which may be due to regional climate dynamics and land surface properties (Seto & Shepherd, 2009). Vegetation variables reinforce the differences: leaf area index (LAI) is higher in NC areas due to reduced urban vegetation (Ordway et al., 2017), while surface net solar radiation (SR) is elevated in CH areas, indicating greater absorption by artificial surfaces. Soil temperature and volumetric soil water content are also higher in NC areas, reflecting more robust land–atmosphere interactions. Population density is significantly higher in CH areas, confirming urban concentration, but the Water Stress Index (WSI) is notably greater in NC areas, suggesting heightened water demand across these landscapes. This indicates that regional climate variations, land degradation, and shifting hydrological patterns are also significant factors driving environmental change. While the boxplots offered initial insights into the variability of key variables, conducting a variable importance analysis through machine learning models allows us to more precisely identify and quantify the factors that significantly influence urban change. This approach builds on the trends suggested by the boxplots, providing a deeper understanding of the relationships between variables and their impact on land use patterns in the KRB.

### Key drivers of urban change across machine learning models

**Fig. 4:**
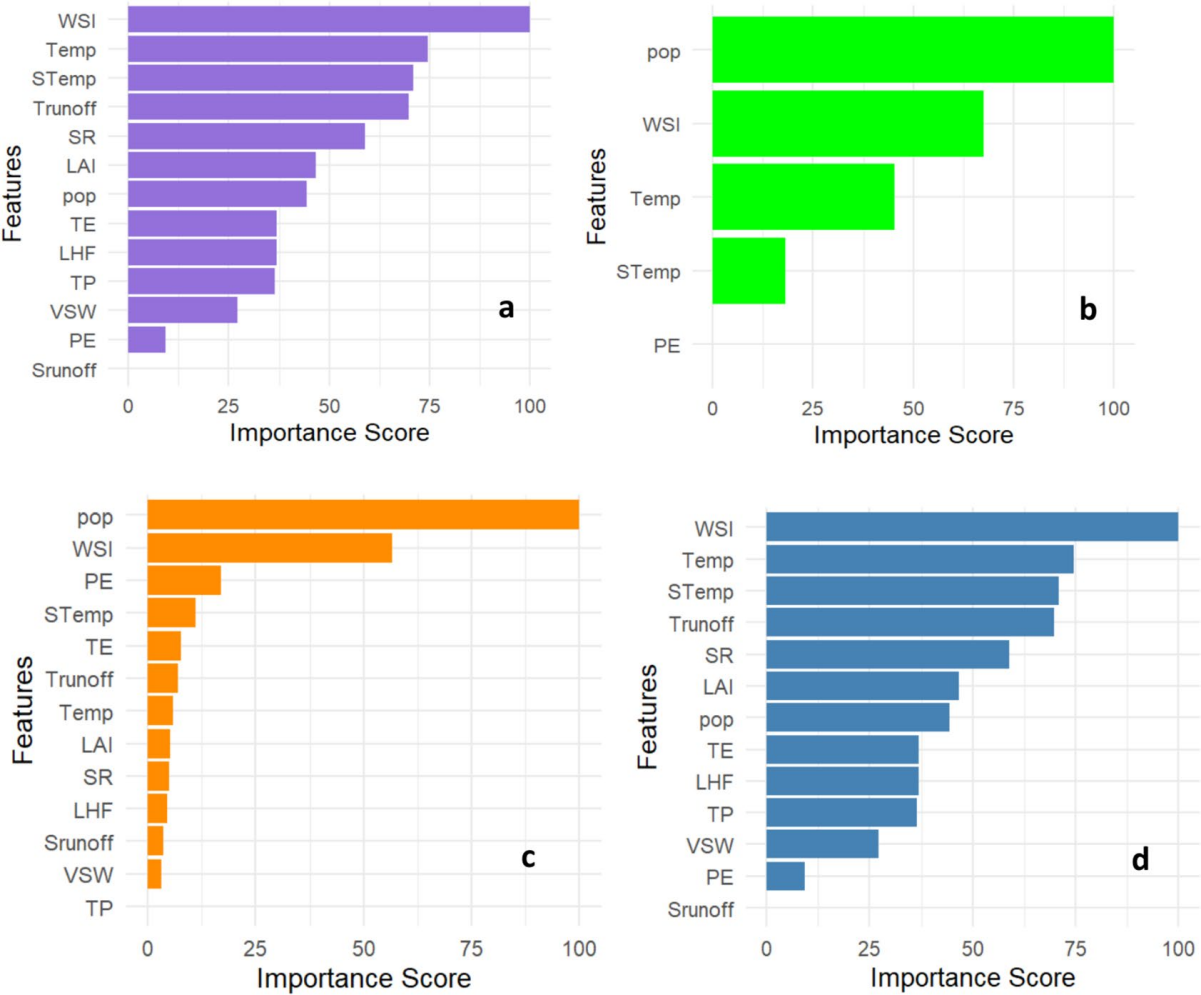
Variable importance across **a **KNN, **b **MARS, **c **RF, and **d** SVM

The variable importance scores from the machine learning models provide valuable insights into the key factors driving urban change in the Kaduna River Basin (KRB) (see Fig. 4). Across all models, population density and the Water Stress Index (WSI) consistently emerge as significant predictors, highlighting these factors’ crucial role in shaping land use changes. This finding aligns with existing literature, which indicates that urbanization and population growth lead to shifts in land cover as more land is converted to meet the growing needs of communities (Waheed et al., 2010; Okafor & Ogbu, 2018; Durowoju, 2021). WSI is especially important in models like KNN and support vector machines (SVM), emphasizing the significance of water availability in influencing urban change. In the context of the KRB, which experiences recurring water stress due to variable rainfall and seasonal droughts, water scarcity and excessive flooding can compel urban areas to adapt their land use (Durowoju et al., 2021b; Isa et al., 2023). This explains why the WSI is prioritized in these models. Research supports this finding by illustrating how water stress often drives land use transformations as cities implement adaptive strategies like infrastructure relocation or water conservation practices (Ahuchaogu et al., 2022).

Temperature and soil temperature consistently rank highly, particularly in the KNN and random forest (RF) models (Fig. 4). This reflects the growing recognition of temperature fluctuations as significant drivers within the KRB. As climate change leads to increased temperatures, the basin is likely to experience more pronounced changes in land cover, including vegetation loss and the expansion of urban heat islands. This further accentuates the need for sustainable urban planning (Grimmond, 2007). Precipitation also appears important, particularly in the multivariate adaptive regression splines (MARS) and RF models, indicating that rainfall patterns significantly impact urban changes. In the KRB, irregular precipitation and extreme rainfall events often lead to changes in land use, especially in flood-prone areas or those transitioning to urban settlements (Durowoju, 2021). Urban sprawl tends to occur around these flood-prone areas, necessitating land use and infrastructure development adjustments to address water-related challenges (Durowoju, 2021). Moreover, runoff and solar radiation play significant roles in the models, especially in KNN, RF, and SVM. These variables underscore the complex relationship between water management and environmental exposure in urban settings. Excessive runoff in the KRB can result in increased flooding, land degradation, and a pressing need for more robust infrastructure development. Similarly, solar radiation influences vegetation growth and energy consumption patterns within urban areas, affecting land use decisions and land cover changes (Santamouris, 2014).

The results from the machine learning models indicate that while demographic factors like population growth are crucial to urban change in the KRB, environmental stressors such as water scarcity, temperature fluctuations, and extreme weather also significantly influence land use patterns. This aligns with broader research on urban transformation, highlighting the interaction between human and environmental factors. We assessed model accuracy with training and test data to validate these findings. This step was essential for ensuring reliability and generalizability. By employing model tuning and cross-validation, we optimized hyperparameters and reduced the risk of overfitting, leading to a more accurate performance estimate on unseen data. The improvements in model performance further reaffirm the importance of the identified variables. Thus, our conclusions regarding the key drivers of urban change in the KRB are supported by thorough statistical validation, reinforcing the role of both human and environmental factors in shaping land use and cover changes within the basin.

### Accuracy of machine learning models

To further understand the ongoing transformation within the KRB and the shifts in LULC, we employed machine learning models to assess urban change predictions (CH and NC) (Table 2). The performance of the four algorithms was evaluated using metrics such as overall accuracy (OA), kappa, F1 score, and area under the curve (AUC). These models help us better understand the dynamics of urban changes, and possible prediction using key factors driving urbanization and land cover changes in the KRB.

**Table 2:** Accuracy of learning algorithms on urban change

Learning algorithms	Sample size	Prediction	Overall accuracy (OA)	Kappa	F1 score	AUC
KNN	57	**CH**	0.72	0.45	0.58	0.60
		**NC**				
MARS	57	**CH**	0.73	0.46	0.76	0.74
		**NC**				
RF	57	**CH**	0.80	0.60	0.74	0.73
		**NC**				
SVM	57	**CH**	0.71	0.42	0.70	0.73
		**NC**				

**Fig. 5:**
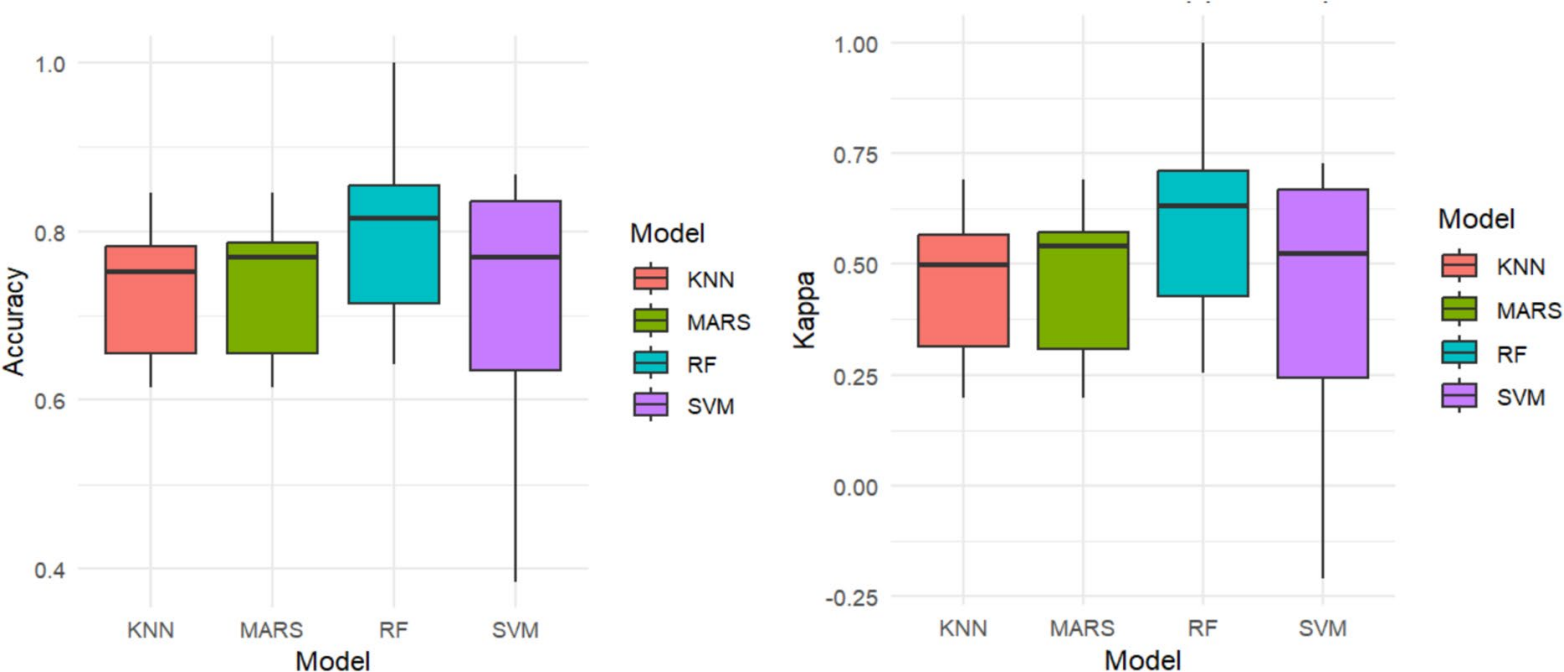
Plots showing accuracy and kappa for the test data

The results from Table 2 show that RF achieves the highest OA at 80%, followed by MARS with 73%, while KNN and SVM have lower accuracy at 72% and 71%, respectively, highlighting RF’s reliability for this task (Fig. 5). As an ensemble method, RF combines multiple decision trees, effectively reducing overfitting and handling complex, high-dimensional data (Breiman, 2001). MARS also performs well, with high F1 scores (0.76) and a strong AUC of 0.74. Its flexibility in modelling nonlinear relationships makes it effective for detecting urban change, supporting findings from previous studies (Oludapo et al., 2022; Orimoloye et al., 2022). However, KNN and SVM show weaker performance, likely due to their sensitivity to data distribution. KNN relies on distance calculations, which can suffer in class overlap or noise (Cover & Hart, 1967). Although powerful in high-dimensional spaces, SVM struggles with noisy or nonlinearly separable data (Cortes & Vapnik, 1995). The kappa statistic, which measures inter-rater agreement, further highlights RF’s superior performance, with a kappa of 0.60, indicating substantial agreement between predicted and actual values (Fig. 5). MARS follows with a kappa of 0.46, reflecting moderate agreement, while KNN and SVM have the lowest kappa values (0.45 and 0.42), suggesting a less consistent classification (Fig. 5). The F1 score shows that MARS and RF strike the best balance between precision and recall, with scores of 0.76 and 0.74, respectively. KNN and SVM perform worse, with scores of 0.58 and 0.70, respectively. The AUC values of RF and MARS (0.73 and 0.74) indicate strong discrimination power between the classes. Although SVM has a comparable AUC (0.73), its accuracy is lower, and kappa suggests it is less reliable than RF and MARS for this task (Fig. 5).

### Spatiotemporal pattern of land use/land cover changes

**Fig. 6:**
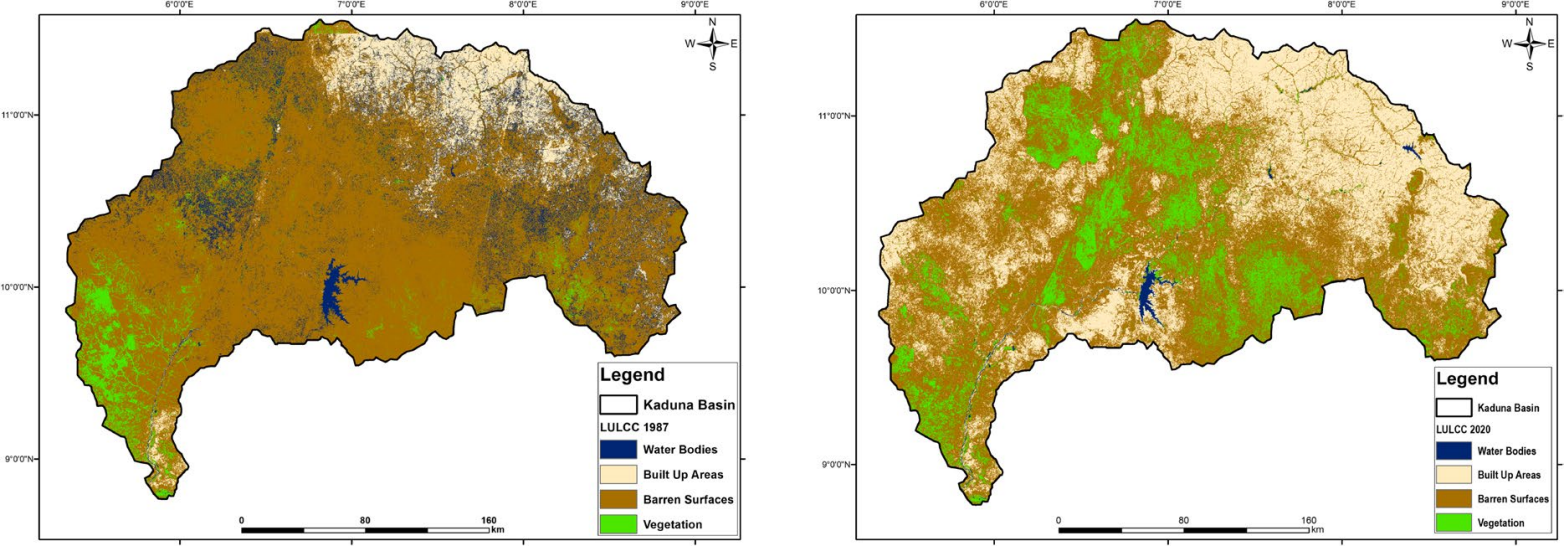
Land use/land cover pattern of Kaduna basin in 1987 and 2020

The changes in LULC in the KRB between 1987 and 2020 (see Fig. 6 and Table 3) are driven by natural processes and human activities (Fig. 4). Over the 33 years, barren surfaces, once the dominant land cover type, saw a substantial decline, reducing from 68.84% (45,348.19 km^2^) to 50.82% (33,479.94 km^2^). This reduction of 26.17% was accompanied by a dramatic 154.81% increase in built-up areas, from 4.26% (9395 km^2^) to 36.34% (23,939.69 km^2^). Vegetation also experienced a positive shift, growing by 174%, from 4.50% (2961.32 km^2^) to 12.32% (8114.10 km^2^). In contrast, water bodies suffered a striking decrease of 95.79%, shrinking from 12.41% (8173.48 km^2^) to just 0.52% (344.26 km^2^).

**Table 3:** Land use/land cover change of Kaduna basin in 1987 and 2020

LULCC	1987				2020		Net change (%)
	Pixels	km^2^	%	Pixels	km^2^	%	
Water bodies	943,993	8173.48	12.41	40,878	344.26	0.52	−95.79
Built-up areas	1,085,072	9395	14.26	2,842,611	23,939.69	36.34	154.81
Barren surfaces	5,237,472	45,348.19	68.84	3,975,426	33,479.94	50.82	−26.17
Vegetation	342,017	2961.32	4.50	963,473	8114.10	12.32	174.00

**Fig. 7:**
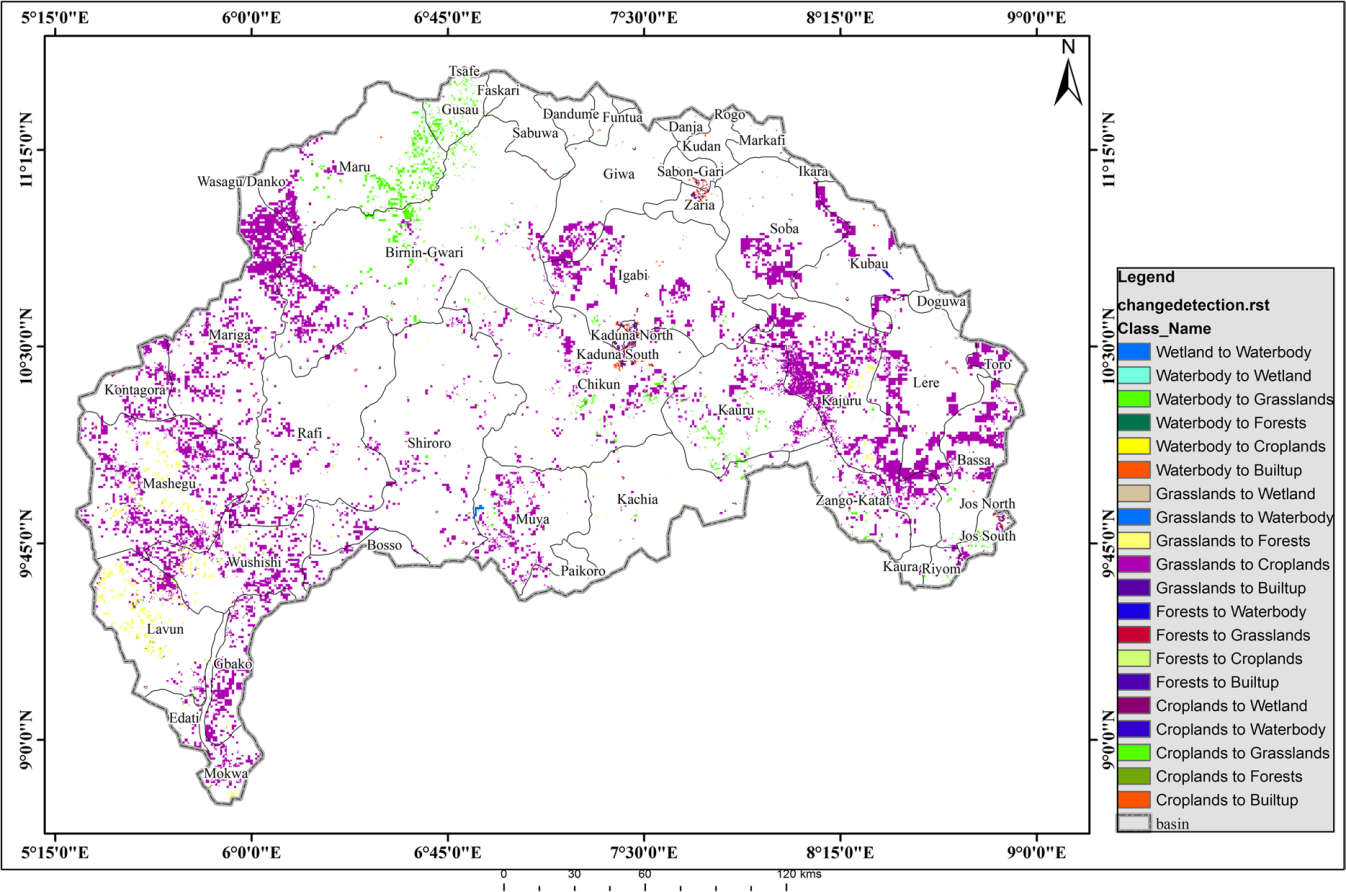
Spatial pattern of landscape conversion between 1987 and 2020

The decline of water bodies and barren surfaces, alongside the growth of built-up areas and vegetation, highlights the region’s dynamic nature of land cover, underscoring the need for informed approaches to water resource management, urban planning, and environmental conservation. Further examination of the land cover changes between the two periods (Fig. 7) reveals that the key transformations primarily involved shifts between wetlands, water bodies, vegetation, barren surfaces, and built-up areas. Notably, wetlands converted minimally into permanent water bodies (12.55 km^2^, 0.019%), a change possibly influenced by seasonal flooding, damming, and hydrological alterations (Durowoju, 2021). The reduction in water bodies transitioning into vegetation (253.45 km^2^, 0.385%) likely resulted from drying, sedimentation, or water diversion, while the marginal conversion of water bodies to built-up areas (4.69 km^2^, 0.007%) signals gradual urban encroachment. The conversion of barren surfaces to built-up areas (6,752.74 km^2^, 10.25%) is the most significant transformation, indicative of rapid urbanization and infrastructure development. In addition, the conversion of barren land to vegetation (482.18 km^2^, 0.732%) suggests a modest increase in vegetative cover, potentially driven by reforestation, afforestation, or natural regrowth (Fig. 7).

## Discussion

### Urban transformation and environmental impacts

Among the metrics considered, the findings highlight the strong capability of RF in monitoring rapid urbanization trends, such as expansion into floodplains and the conversion of farmlands in the Kaduna River Basin, followed closely by MARS. These findings provide valuable insights for guiding urban planning, environmental management, and climate adaptation strategies in the study area to mitigate the loss of vegetation, biodiversity, and water recharge zones. Variable importance analysis reveals that the Kaduna River Basin has undergone significant transformations due to population growth and urbanization, leading to various environmental and socio-economic challenges. Rapid urban expansion has substantially encroached into the river’s floodplain (Durowoju et al., 2021a), reducing its natural capacity to manage floodwaters and increasing the frequency and severity of flooding events (Alayande & Agunwamba, 2010). Moreover, a growing population has intensified pressure on water resources, increasing water stress across the basin.

These findings align with studies highlighting the negative effects of urban growth on water resources in the Kaduna metropolis, emphasizing the urgent need for sustainable urban planning (Muhammad & Onoja, 2023; Ologunorisa et al., 2021). Agricultural lands and natural vegetation have been converted into built-up areas to accommodate the growing population, disrupting local ecosystems, increasing surface runoff, decreasing groundwater recharge, elevating flood risks, and contributing to water scarcity during dry periods (Opatoyinbo et al., 2015). Climate change further exacerbates these challenges, significantly altering climate extreme indices in the region, affecting water availability and increasing flood frequencies. The basin’s sensitivity to climate variability necessitates adaptive water management strategies to mitigate these negative impacts on agriculture and water supply (Isa et al., 2023). The water stress index (WSI) results indicate a demand–supply imbalance in the region’s water resources, influencing land use practices. Water scarcity drives adaptive land transformations, such as alterations in cropping patterns or irrigation infrastructure (Durowoju et al., 2023) and worsening water stress, particularly during low rainfall periods. A recent study assessing land use changes in Kaduna reported a 145% increase in urban areas between 2001 and 2014, primarily in the southern metropolis (Kafi et al., 2024). This urban sprawl leads to incompatible land uses and places additional pressure on water supplies. The expansion of irrigated farming practices further strains water resources, resulting in over-extraction from rivers and groundwater sources, potentially reducing water availability for other uses (Daramola et al., 2022; Durowoju et al., 2023; Ologunorisa et al., 2021).

### Hydroclimatology and urban transformation

The implications of hydroclimatological conditions on urban changes are profound, affecting cities’ sustainability, livability, and resilience. Urban areas experience higher temperatures than rural areas due to the urban heat island (UHI) effect (Fashae et al., 2020a, 2020b; Durowoju et al., 2021c). This phenomenon is driven by heat-absorbing materials like concrete and asphalt, limited vegetation, and concentrated human activities. As cities expand, the effects of UHI intensify, increasing energy consumption for cooling, heat-related illnesses, and shifts in local weather patterns (Anibaba et al., 2017, 2019; Durowoju et al., 2021b). Urbanization significantly alters natural water cycles. The construction of impermeable surfaces increases surface runoff and reduces groundwater recharge (McGrane, 2016), leading to flooding, water pollution, and the degradation of aquatic ecosystems (Chakraborty & Chakraborty, 2021).

A net reduction in water bodies results from expanding built-up areas and irrigation farming (Babade et al., 2022), which stresses natural resources, particularly on barren surfaces (Hassan et al., 2016). The conversion of barren and natural lands into built-up areas was significant during the study period. Urban expansion, driven by economic opportunities, contributes to vegetation degradation (Obateru et al., 2024). If the ongoing environmental degradation persists, the region may experience heightened surface temperatures and soil erosion (Azadi et al., 2018), exacerbating concerns about food and water availability while amplifying global warming (Kogo et al., 2021). Climate and land use changes have increased temperatures and reduced precipitation, leading to wetland drying, declining water resources, and impaired biogeochemical processes crucial for crop productivity (Msofe et al., 2019; Obateru et al., 2023). Spatially, built-up areas expanded notably in the northeastern part of the study area, extending into major cities (Fig. 7). The wetlands, predominantly occupied by farming communities, have undergone significant reductions, with water bodies shrinking from 12.41 to 0.52%, largely due to over-extraction from the Shiroro Reservoir (constructed in 1990) and increased reliance on irrigation from the River Kaduna and its tributaries. Key predictors of urban change in the region include population, water stress index, and soil temperature (Fig. 4). Given climate change and human activities, urban transformations are expected to persist. A significant decline in water bodies suggests a potential shift toward hydrological drought, escalating into agricultural drought over time (Babade et al., 2022; Durowoju et al., 2022; Orimoloye et al., 2022). Persistent change detection results indicate that increasing built-up areas lead to higher runoff, surface temperatures, and intensified urban heat island effects (Anibaba et al., 2017, 2019; Eresanya et al., 2019; Ologunorisa et al., 2021).

Furthermore, urban expansion also diminishes the basin’s ability to supply ecosystem services such as air purification, soil erosion protection, heat mitigation, noise regulation, wildlife habitat, and human-nature interactions (Obateru et al., 2024). The conversion of water bodies into vegetation reflects reduced water availability due to climate change, prolonged droughts, increased sediment deposition, and human activities like irrigation withdrawals and dam regulations that alter hydrological patterns. Wetland loss significantly reduces the basin’s capacity for water storage and purification. A geospatial analysis of Kaduna’s land use changes from 1986 to 2023 revealed substantial wetland losses, including a 15 km^2^ decline in marshlands and a 28.6 km^2^ reduction in riparian vegetation (Abubakar & Abdussalam, 2024). Such changes highlight the need for sustainable water and land management strategies to ensure the resilience of the Kaduna River Basin against climate and anthropogenic pressures.

## Conclusion

This study demonstrated the effectiveness of integrating Google Earth Engine (GEE) and machine learning (ML) algorithms in analyzing urban change dynamics. The results confirm that urban growth is intricately linked to shifts in key biophysical variables such as population density, water stress index, leaf area index, precipitation, total and surface runoff, potential evapotranspiration, and soil temperature, highlighting the complex interplay between climate, land cover, and urbanization. Notably, the findings showed significant reductions in barren surfaces (26.17%) and water bodies (95.79%) over three decades, emphasizing the profound environmental transformations occurring within the study area. The model performances, with overall accuracies of 0.80 (RF), 0.73 (MARS), 0.71 (SVM), and 0.72 (KNN), further reinforce the reliability of ML-based urban change detection.

Beyond its methodological contributions, this study underscores the need for sustainable urban planning strategies. Cities must adopt solutions that can mitigate the impact of the changing climate and enhance climate resilience. Integrating sustainable drainage systems and green infrastructure will also be critical for stormwater management, water quality preservation, and urban biodiversity enhancement.

While these findings offer valuable insights, they also open avenues for future research. Future studies could explore the socio-economic implications of urban expansion, assessing how land-use changes impact livelihoods, public health, and social equity. Additionally, incorporating higher-resolution satellite data and deep learning techniques could improve model precision and extend applications to other rapidly urbanizing regions. Understanding how urbanization-induced environmental changes interact with extreme climate events such as heatwaves and flooding will also be essential in shaping adaptive urban policies. Ultimately, continued advancements in remote sensing, geospatial analytics, and machine learning will be crucial for building sustainable, climate-resilient cities in an era of accelerating global change.

## Data Availability

No datasets were generated or analysed during the current study.
